# The Genetics of Atypical Femur Fractures—a Systematic Review

**DOI:** 10.1007/s11914-021-00658-y

**Published:** 2021-02-15

**Authors:** Wei Zhou, Jeroen G. J. van Rooij, Peter R. Ebeling, Annemieke J. M. H. Verkerk, M. Carola Zillikens

**Affiliations:** 1 Department of Internal Medicine, Erasmus University Medical Center, Rotterdam, the Netherlands; 2 Department of Neurology & Alzheimer Center, Erasmus University Medical Center, Rotterdam, the Netherlands; 3 Department of Medicine, School of Clinical Sciences, Monash University, Clayton, Australia

**Keywords:** Atypical femur fractures, Genetics, Osteoporosis, Bisphosphonates

## Abstract

**Purpose of Review:**

Atypical femur fractures (AFFs) are rare subtrochanteric or diaphyseal fractures regarded as side effects of bisphosphonates (BPs), possibly with a genetic background. Here, we summarize the most recent knowledge about genetics of AFFs.

**Recent Findings:**

AFF has been reported in 57 patients with seven different monogenic bone disorders including hypophosphatasia and osteogenesis imperfecta; 56.1% had never used BPs, while 17.5% were diagnosed with the disorder only after the AFF. Gene mutation finding in familial and sporadic cases identified possible AFF-related variants in the *GGPS1* and *ATRAID* genes respectively. Functional follow-up studies of mutant proteins showed possible roles in AFF. A recent small genome-wide association study on 51 AFF cases did not identify significant hits associated with AFF.

**Summary:**

Recent findings have strengthened the hypothesis that AFFs have underlying genetic components but more studies are needed in AFF families and larger cohorts of sporadic cases to confirm previous results and/or find novel gene variants involved in the pathogenesis of AFFs.

**Supplementary Information:**

The online version contains supplementary material available at 10.1007/s11914-021-00658-y.

## Introduction

An atypical femur fracture (AFF) is a rare, low-trauma fracture occurring at the subtrochanteric or diaphyseal area of the femur [1]. The peculiarity of AFF lies in its transverse morphology, and the fact that it occurs at the strongest part of the femur without high-impact trauma. This distinguishes it from classic osteoporotic fragility fractures that occur at the intertrochanteric femur or the femoral neck. AFFs may be either complete or incomplete. They were first recognized as a potential rare side effect of bisphosphonates (BPs) in the first report of AFFs in 2005 [2].

The American Society of Bone and Mineral Research (ASBMR) published a report on AFF case definition [1, 3]. In the most recent diagnostic criteria (2014), an AFF should fulfill at least four of five major features: (1) minimal or no trauma; (2) originates at the lateral cortex and has transverse orientation; (3) non-comminuted or minimally comminuted; (4) complete fractures extend through both cortices and incomplete fractures involve only the lateral cortex; (5) localized cortical thickening at the fracture site [1].

Concern caused by case reports and epidemiological studies pointing toward an association between BPs and AFF has resulted in a dramatic 50% decrease in the use of BPs for the prevention of osteoporotic fractures [4–8]. The incidence of AFF is between 1.1 and 9.8 per 100,000 person-years; however, it increases 2–3 times after every 2 years of BP use and rises up to 112–131 per 100,000 person-years for more than 8 years of BP use [9–13]. Interestingly, 10–50% of AFF occur in BP-naïve patients [10, 11, 14, 15]. Additional factors increasing AFF risk are Asian ethnicity, greater femoral bowing, use of glucocorticoids, lower height, and higher body weight [12, 16, 17•, 18]. Other suggested but less well understood risk factors are rheumatoid arthritis, diabetes mellitus, and use of proton pump inhibitors [19]. AFFs occur more frequently in women, possibly due to higher rates of BP use [20].

The etiology of AFF is still unclear. It has been suggested that AFFs are stress fractures caused by normal loading on bone that has an abnormal structure or increased brittleness. They initiate from the lateral cortex that sustains high tensile stress levels and develop as microdamage accumulates [1]. Factors such as impaired bone quality or impaired microcrack repair may contribute to the fracture occurrence. BPs may contribute because their use may lead to more homogeneously mineralized bone, accumulation of advanced glycation end products (AGEs), and suppressed bone turnover and hence they may reduce the quality and the ability to repair microdamage [21–24].

### Previous Evidence for Genetic Factors of AFFs

Although rare, familial aggregation of AFFs and the occurrence of AFF in patients with a monogenetic bone disorder, suggest a genetic component, as reviewed previously [25•]. AFF has been reported in hypophosphatasia (HPP), X-linked hypophosphatemia, pycnodysostosis, osteopetrosis, osteoporosis pseudoglioma syndrome (OPPG), osteogenesis imperfecta (OI), and X-linked osteoporosis, involving genes such as *COL1A1*, *COL1A2*, *ALPL*, *PHEX*, *CTSK*, *LRP5*, *SERPINF1*, *IFITM5*, *CRTAP*, and *PLS3* [25•]. Pérez-Núñez et al. first associated several genetic variants with AFF through exon-wide analysis of 13 women with AFF and 268 control women with or without osteoporosis [26], although the relevance of those variants for AFF remained unclear. Bhattacharyya et al. could not associate coding variants in *ALPL* with AFF in 9 patients and 13 controls with mean BP use of 9 years [27]. Roca Ayats et al. identified shared variants by exome sequencing of three sisters with AFF in *GGPS1*, *MVD*, and *CYP1A1*, along with other genes [28••].

To further expand the knowledge on the genetic background of AFF, we systematically reviewed relevant papers and conference abstracts published since the previous review in 2017. We refer the readers to the Appendix for the methods, including a flow diagram of search results. As many gene names are mentioned in this review as an abbreviation, a list of full gene names can also be found in the Appendix.

## Results

We updated the table summarizing the reports of AFF in monogenic bone disorders by Nguyen and van de Laarschot et al. with newly published cases in Table 1. In addition to the previously reported 23 cases of AFF in patients with seven different types of monogenic bone diseases, including 4 HPP patients and 5 OI patients, recent publications have reported 19 additional AFF cases with HPP and 15 with OI.

**Table 1:** List of monogenic bone disorders associated with atypical femur fractures (AFFs)

Monogenic bone disorder	Genes reported	Gender	Age (years)	Bilateral AFFs (*N*)	BP exposure (*N*)	Disorder diagnosed after AFF (N)	References
Hypophosphatasia	*ALPL*	20 F + 3 M	36–76	15	10	6	[25•, 29–32]
X-linked hypophosphatemia	*PHEX*	1 M	27	0	0	0	[25•]
Pycnodysostosis	*CTSK*	3 M + 4 F	23–55	3	0	2	[25•]
Osteopetrosis	None	4 F	21–56	2	0	1	[25•]
OPPG	*LRP5*	1 M	38	0	0	1	[25•]
Osteogenesis imperfecta	*COL1A1*, *COL1A2*, *SERPINF1*, *IFITM5*, *CRTAP*	13 F + 7 M	0.2–75	6	14	0	[25•, 33, 34, 35•, 36•]
X-linked osteoporosis	*PLS3*	1 M	18	0	1	0	[25•]
Total		57		26	25	10	
Percentage		100%		45.6%	43.9%	17.5%	

Notes: The table is adapted from Nguyen and van de Laarschot et al. [25•] and updated with new cases

*AFF* atypical femur fracture; *N* counts; *BP* bisphosphonate; *OPPG* osteoporosis pseudoglioma syndrome

### AFFs Reported in Monogenic Bone Disorders—HPP

Lawrence et al. and Righetti et al. each reported a HPP patient carrying a heterozygous ALPL variant with bilateral AFFs preceding the diagnosis, one without known osteoporosis or BP use, and one having used BPs for 10 years, respectively [29, 30]. Lefever et al. identified two out of 14 HPP patients with AFFs, one of them after BP use, both carrying variants in *ALPL*, one homozygous and one heterozygous [31]. A cohort study of 150 HPP patients described the characteristics of 25 subtrochanteric and/or diaphyseal femoral fractures occurring in 15 patients [32], an incidence of 10%. Twenty-two of these fractures met the criteria for AFF, while the three others initiated from the medial cortex. Six out of these 15 patients had previously been treated with BPs. Among 14 patients with genotyping data, all were reported to carry homozygous or compound heterozygous variants in *ALPL*.

### AFFs Reported in Monogenic Bone Disorders—OI

Several new studies have described the occurrence of AFFs in patients with OI. Tan and Seow, and Chen et al. each reported an OI patient with an AFF after 9 and 10 years’ BP use, respectively, both without an identified genetic cause [33, 34]. Anderson et al. evaluated 55 OI patients and identified eight patients having suffered femoral fractures, of which four met the criteria for AFF, three of them also had severe varus deformity of the femur [35•]. All four patients had been exposed to BPs. Missense variants in *COL1A1* and *COL1A2* were identified in two of the patients. Trejo et al. identified 11 AFFs in 691 children with OI, of whom eight were BP-naïve [36•]. The study excluded OI patients with femur deformities for AFF identification and found that AFF occurrence was associated with the severity of OI rather than BP use. In another study examining femoral fractures in OI patients, those with and without BP treatment showed similar femoral fracture characteristics with transverse and supracondylar femoral fractures being more frequent in OI type III and IV compared with OI type I [37].

In total, 57 AFF patients with seven monogenic bone disorders have been reported, 32 (56.1%) of whom without prior use of BPs and 10 (17.5%) where the diagnosis of the bone disorder was only made after the AFF (Table 1).

### AFF Occurring in Genetic Muscle Disease

Spinal muscular atrophy (SMA) is an autosomal recessive disorder characterized by degeneration of lower motor neurons and progressive atrophy of skeletal muscles caused by variants in the survival motor neuron 1 (*SMN1*) gene. In a study assessing the safety and efficacy of intravenous BPs in children with SMA at high risk for low bone mineral density and fragility fractures, one AFF was observed in 32.7 patient-years of follow-up [38]. This patient suffered 6 unspecified long bone fractures prior to BP use, and did not have any additional fractures during 3 years of exposure to BPs before the AFF occurred after low trauma. Based on a single event, it is unclear whether the underlying genetic condition causing the muscular atrophy was related to the AFF or not.

### Family Studies on AFF

Roca Ayats et al. identified 37 variants in 35 genes shared by three sisters with osteoporosis, AFF, and more than 5 years of BP use [39]. Among them, the *GGPS1* variant resulting in the p.D188Y substitution in GGPPS (Table 2), an enzyme in the mevalonate pathway, was studied in detail [42••]. GGPPS enzyme activity was reduced in p.D188Y mutant *E. coli* cells [42••]. ShRNA-mediated knockdown of *GGPS1* in osteoblasts reduced mineralization and expression of osteocalcin (BGLAP), osterix (OSX), and receptor activator of nuclear factor kappa-Β ligand (RANKL). A non-significant decrease in resorption activity was observed in osteoclasts [42••]. Lisnyansky et al. further showed that D188Y interfered with substrate binding to GGPPS and decreased catalytic activity fourfold [43••]. Furthermore, D188Y mutant GGPPS demonstrated minimal enzyme activity in the presence of the BP, zoledronic acid, and partially decreased binding affinity with zoledronic acid compared with wild-type GGPPS. The authors proposed that zoledronic acid further hampered activity of D188Y mutant GGPPS to an extent below the threshold level to maintain osteoclast function. However, until pathogenic *GGPS1* variants are identified in other AFF patients, the extent of *GGPS1* contribution to AFF is uncertain. Among the other 34 genes identified by Roca Ayats et al., 12 were described to have functions possibly related to AFF, such as *FN1*, which encodes for fibronectin, and *CYP1A1*, which is involved in drug metabolism, and the authors raise the hypothesis of a polygenic nature of AFF, which currently remains untested. Additional variants of uncertain significance have been identified in genes such as *MVD*, *CYP1A1*, and *RUNX2* in unrelated AFF patients, although currently unsupported by functional assessment or case-control analysis [39].

**Table 2:** Summary of published variants associated with AFF

Gene	Variant	rs-number	Effect on the protein	Reference
*GGPS1*	chr1: g.235505746G > T		p.D188Y	[39]
*OFD1*	Not specified		p.A642D	[40]
*ATRAID*	chr2: g.27435250A > G	rs1275533	p.D5G	[41••]

Note: the genome positions are in Genome Reference Consortium Human Build 37 (hg19)

Boisvert et al. described in an abstract, a family in which 11 relatives have X-linked osteoporosis, and/or dental and facial hypoplasia and/or adult-onset multiple stress fractures of long bones, with radiological appearances akin to AFF [40]. A variant in the centriole and centriolar satellite protein gene (*OFD1*) resulting in the p.A642D substitution in the protein (Table 2) was reported as the only candidate perfectly segregating with the phenotypes after exome sequencing analysis. BP use for this family was not reported. Sequencing of *OFD1* in 49 additional AFF patients who did use BPs, identified another variant in one patient, resulting in a p.K668N substitution. *OFD1* is associated with oral-facial-digital syndrome type I and is important for the development of many parts of the body by regulating Wnt signaling [44]. Until proven by functional assays and replication of these results, it is unknown whether the variants in this gene cause these bone phenotypes and whether and how they are related to AFF.

### Basic Research and Candidate Gene Studies in AFF

In order to gain insight into the mechanisms and genes that are required for the action of nitrogen-containing-bisphosphonates (N-BPs), Yu et al. performed a CRISPRi-mediated genome-wide screen in cancer cells and in this way identified the solute carrier family 37 member 3 gene (*SLC37A3*) as altering responses to alendronate [45]. SLC37A3 is involved in transporting N-BPs into osteoclasts and its function is dependent on ATRAID, which was previously identified as a genetic target of N-BPs [45]. In addition, ATRAID-deficient osteoporotic mice showed resistance to the therapeutic effect of alendronate [41••]. As a follow up of this study, exome-sequencing data in patients taking N-BPs and showing side effects like AFF and osteonecrosis of the jaw was investigated for rare nonsynonymous coding variants. This resulted in the identification of a p.D5G substitution in the ATRAID protein (Table 2) in two out of 27 AFF patients, as compared to 1.3% in the general population. It was found that D5G mutant ATRAID conferred hypersensitivity to alendronate compared with wild-type ATRAID in HEK-293 T cells [41••]. By combining the results from the WES data with the CRISPRi screening data and gene expression data from BP users, Surface et al. identified two other genes, *ATR* and *ZBTB4*, as possible regulators in N-BP response.

Candidate gene screening in AFF patients has identified rare variants in *COL1A2* [46], *CYP1A1* [47], and *ALPL* [47]. While rare variants in *COL1A1* and *ALPL* may confer mild forms of monogenic bone disorders and are related to the occurrence of AFF, variants in *CYP1A1* cannot be confirmed to be causal to AFF unless a significant association is observed in case-control analysis or until the variants have been confirmed as pathogenic in functional experiments.

### Genome-Wide Association Study in AFF

A recent genome-wide association study (GWAS) was conducted on 51 AFF patients treated with BPs compared to 324 controls matched on sex and BP use, as well as to 8709 population controls [48••]. Four uncommon single-nucleotide polymorphisms were associated with AFF when compared with population controls, which are near *NR3C1* and the pseudogene *TUBB8P5* and in intronic regions of *IL18R1* and *NTN1* respectively, but these signals were insignificant when compared with BP-matched controls. A few loci, presented with small *p* values, near genes such as *GALNS* and *CNTN4* may be suggestive, but larger GWAS studies are needed to confirm any of these findings.

## Discussion

In this review, we made an inventory of genetic factors described in AFF patients since our previous systematic review in 2017 [25•]. In addition to publications from earlier years, we describe additional AFF patients with monogenic bone disorders, including HPP and OI, now totaling 57 published cases in seven diseases, of which more than half had no prior BP use. Ten of these cases were diagnosed with the monogenic bone disorder only after having sustained the AFF. Notably, these monogenic disorders affect different aspects of bone physiology, including bone mineralization, collagen synthesis and structure, bone remodeling, and osteocyte function [25•]. We might hypothesize that carriers of variants in the genes associated with monogenic bone disorders presenting with mild forms of these diseases may be at increased risk for AFF, while the role of BPs in contributing to AFF in these disorders remains to be determined. However, BPs could be seen as a second factor impairing repair of microfractures, e.g., by decreasing bone turnover and/or leading to a more homogeneously mineralized bone matrix, less resistant to microcrack propagation. No novel bone disorders were associated with AFF, although for the first time, AFF was reported in a patient with spinal muscular atrophy treated with BPs [38]. A causal relation with the genetic variant causing SMA or with the muscle disease itself is currently unclear.

Exome sequencing in three sisters with AFFs previously identified variants in 35 genes. Among these, *GGPS1* was the most likely candidate for the genetic cause of AFF, supported by functional assays of the p.D188Y substitution resulting in decreased GGPPS activity, which was further suppressed by BPs [43••]. Two additional AFF candidate genes were identified, including an *ATRAID* variant resulting in p.D5G substitution in two of 27 AFF patients, which increased sensitivity to alendronate in HEK-293 T cells [41••] and possibly *ODF1* in which two different variants were described, one segregating in a family with a phenotype including multiple stress fractures of long bones (p.A642D) and one in a sporadic case with AFF and BP use (p.K668N) [40]. Further, a recent GWAS performed in AFF patients using different control groups did not show genome-wide significant results when the analysis was controlled for BP use [48••]. Earlier exon-wide and genome-wide studies on AFF in 13 and 51 cases, respectively, suggested the possibility of a polygenic architecture of AFF [26, 48••]. None of these results has been fully confirmed, mainly because of the limited power due to the rarity of AFF and hence the small sample size of the cases studied.

The variation of AFF-related genes, across many bone metabolism pathways and monogenic bone disorders suggests a heterogeneous background. The mevalonate pathway is the target of nitrogen-containing BPs, but AFFs have also been observed in patients treated with denosumab, which is a RANKL inhibitor, preventing development of osteoclasts [49]. The study conducted by Surface et al. is pointing toward genetic variants related to BP transport/hypersensitivity, events occurring upstream of farnesyl diphosphate synthase (FDPS) in the mevalonate pathway [41••]. These observations suggest that different genes related to different molecular pathways may influence the sensitivity to bone active drugs or may increase AFF susceptibility independent of these drugs.

Larger cohort or case-series studies are now needed to confirm the above results and uncover robust underlying mechanisms. For rare diseases like AFF, this can only be achieved by international collaboration, collecting cases from different countries. However, combining all these cases with different ethnicities would also require specific control groups to be carefully selected.

In studying the genetics of AFF, gene-based or pathway-based analysis in sporadic cases that examine the burden of rare variants in certain genes or pathways can be applied to increase power. Below we discuss some important considerations for future AFF genetic study designs.

### The Role of Ethnicity and Bone Geometry

Several studies report a higher incidence of AFF in Asian ethnicity [17, 50], which has been mainly attributed to differences in femoral geometry, including more prominent femoral bowing in Asians, especially in women [51]. No studies examining genetic influences on AFF have been performed in Asians, however, it is possible that the higher incidence of AFF in Asians may be associated with genetic variants related to femoral geometry.

Genetic factors related to either subtrochanteric AFF or mid-shaft AFF may be different. Previously, it was reported that AFFs occurred predominantly at the diaphyseal region in the European population, while in the Asian population, they occurred mainly at the subtrochanteric region [52]. However, in Korea and Japan, diaphyseal AFFs were reported as commonly as subtrochanteric AFFs [53, 54]. Both studies found that the degree of femoral bowing is greater in mid-shaft AFF compared with subtrochanteric AFF. Oh et al. proposed that the majority of the mid-shaft AFFs are merely stress fractures due to femoral deformity, while subtrochanteric AFFs and a small percentage of mid-shaft AFFs are caused by severely suppressed bone turnover induced by BP use [55].

### The Role of Osteoporosis and Bisphosphonates

Osteoporosis is a common multifactorial disease caused by interactions of environmental factors and genetic components. Hundreds of susceptibility loci have been identified for bone mineral density through GWAS [56]. Most AFFs have been identified in patients with osteoporosis and some of them used other osteoporosis treatment than BPs, such as denosumab [49, 57], which may be related to either the later introduction and lower use of denosumab, or to differences in absolute risk for AFF between the two drug classes, which is currently unknown. While genetic factors contributing to osteoporosis may confound genetic studies of AFF, it is possible that these factors also contribute directly to the pathogenesis of AFF. The complexity of the situation is illustrated by the fact that it is currently unknown if one or more genetic variants related to monogenic bone diseases or osteoporosis increase susceptibility to AFF or if there are (additionally) separate gene variants for AFF and/or if the use of antiresorptive drugs potentiates the effects of these gene variants.

In studying BP-associated AFFs, BPs may be considered a mediator or a modifier in the relation between genetic factors and AFF. Yu et al. found that silencing of *FDPS* and *GGPS1* in mevalonate pathway sensitized cells to alendronate, while silencing of the other genes upstream of the pathway, such as *HMGCS1*, *HMGCR*, *MVK*, and *PMVK*, actually made cells resistant to alendronate [45]. Both Surface et al. and Lisnyansky et al. have suggested hypersensitivity to BPs under therapeutic dosage, with resultant toxicity, as contributing to AFFs [41••, 43••]. The interplay between BP use and genetic variations requires further investigation, but genes such as those identified in these studies are interesting candidates for such genetic investigations. In conducting genetic studies in AFF families, as well as in cohorts of isolated cases it also should always be kept in mind that unaffected individuals normally used as controls could in theory develop AFF if they were treated (for a longer time) with a BP.

Finally, when a gene is identified as a candidate, functional proof is needed by in vitro and/or in vivo experiments to elucidate the functional role in the pathogenesis of AFF.

## Summary and Conclusions

While AFF remains a highly concerning event, considered as a rare complication of BP use, the pathogenesis and relationship of AFFs with BPs are still not fully understood. Moreover, the genetic architecture of AFF is still unknown and likely to be complex, i.e., genetically heterogeneous and interacting with other factors such as medication use. The current review updates the previous evidence suggesting genetic components in the etiology of AFF. The genes that have been implicated in AFF include genes associated with monogenic bone disorders, often without use of antiresorptive drugs, such as, *COL1A1*, *COL1A2*, *ALPL*, *PHEX*, *CTSK*, *LRP5*, *SERPINF1*, *IFITM5*, *CRTAP*, *PLS3*, and *OFD1*, and genes involved in the action of BPs, such as *GGPS1* and *ATRAID*. Although studies have been conducted with genetic analysis in AFF families and genome-wide or exon-wide association analysis in unrelated cases, none of the results from the studies has been fully confirmed, mostly because of limitations in sample size.

This emphasizes the urgent need for genetic studies in larger cohorts of isolated cases with appropriate controls and in more families with AFF, using whole-exome sequencing or whole-genome sequencing analysis followed by functional analyses. Important factors to consider in the genetic architecture of AFFs are potential ethnic differences, the interplay between BPs and genetic variations, the possibly overlapping genetic factors with osteoporosis, and genetic heterogeneity. Studying genetics helps to elucidate the pathogenesis of AFF and improve our understanding of bone biology and diseases, as well as to reassure patients who should be treated with BPs or denosumab. Unraveling the genetic architecture of AFF may also bring us closer to personalized treatment if the susceptibility of a patient to AFF could be determined using genetic screening tools.

## Supplementary Information

ESM 1 (DOCX 116 kb)
