# Supplementary material for: The Genetics of Atypical Femur Fractures—a Systematic Review
Source: Curr Osteoporos Rep. 2021 Feb 15;19(2):123–30. doi: 10.1007/s11914-021-00658-y (PMC8016774; doi:10.1007/s11914-021-00658-y)
Supplement: Supplementary file 1 — (DOCX 116 kb) [file 11914_2021_658_MOESM1_ESM.docx]

**Methods**

We used the following keywords: femur/femoral fracture or subtrochanteric fracture, atypical or drug‐induced disease, BPs, antiresorptives and denosumab for our search in Embase, Medline, Web of Science, Cochrane Central and Google Scholar on July 9^th^, 2020. The exact search terms are listed below.

The search was confined to papers and conference abstracts published in English. The inclusion criteria included: 1) cases of AFFs in monogenic musculoskeletal diseases published in journals, and 2) genetic studies in individuals or families of AFF. Articles were only included if images of the femoral fracture were available and fulfilled the ASBMR case definition or if the recent ASBMR case definition for AFF was used. Records before January 2017 or having been identified in the previous systematic review were excluded.

The search was updated on September 22^nd^, 2020, retrieving 80 additional records, however, none of these met the inclusion criteria.

**Search terms used**

**Embase.com**

('femur fracture'/exp OR (((femur* OR femoral* OR subtrochant* OR atypical*) NEAR/6 fracture*)):ab,ti,kw) AND ('drug induced disease'/de OR 'bisphosphonic acid derivative'/exp OR 'denosumab'/de OR (atypical* OR ((drug OR pharmac* OR agent*) NEAR/3 induc*) OR bisphosphon* OR (anti NEAR/3 (resorptive*)) OR antiresorptive* OR alendron* OR butedron* OR clodron* OR etidron* OR ibandron* OR pamidron* OR risedron* OR zoledron* OR denosumab*):ab,ti,kw) NOT ([Conference Abstract]/lim AND [1800-2017]/py) AND [ENGLISH]/lim

**Medline (Ovid)**

("Femoral Fractures"/ OR (((femur* OR femoral* OR subtrochant* OR atypical*) ADJ6 fracture*)).ab,ti,kf.) AND (exp "Diphosphonates"/ OR "Denosumab"/ OR (atypical* OR ((drug OR pharmac* OR agent*) ADJ3 induc*) OR bisphosphon* OR (anti ADJ3 (resorptive*)) OR antiresorptive* OR alendron* OR butedron* OR clodron* OR etidron* OR ibandron* OR pamidron* OR risedron* OR zoledron* OR denosumab*).ab,ti,kf.) NOT ((news OR congres* OR abstract* OR book* OR chapter* OR dissertation abstract*).pt. AND 1800:2017.(sa_year).)

**Cochrane central**

((((femur* OR femoral* OR subtrochant* OR atypical*) NEAR/6 fracture*)):ab,ti,kw) AND ((atypical* OR ((drug OR pharmac* OR agent*) NEAR/3 induc*) OR bisphosphon* OR (anti NEAR/3 (resorptive*)) OR antiresorptive* OR alendron* OR butedron* OR clodron* OR etidron* OR ibandron* OR pamidron* OR risedron* OR zoledron* OR denosumab*):ab,ti,kw)

**Web of science**

TS=(((((femur* OR femoral* OR subtrochant* OR atypical*) NEAR/5 fracture*))) AND ((atypical* OR ((drug OR pharmac* OR agent*) NEAR/2 induc*) OR bisphosphon* OR (anti NEAR/2 (resorptive*)) OR antiresorptive* OR alendron* OR butedron* OR clodron* OR etidron* OR ibandron* OR pamidron* OR risedron* OR zoledron* OR denosumab*)) )

**Google scholar**

"femur|femoral|subtrochanteric|atypical fracture|fractures" atypical|bisphosphonic|bisphosphonate|"anti resorptive"|antiresorptive|alendronic|alendronate|risedronate|risedronic|zoledronate|zoledronic|denosumab

A total of 1150 records were retrieved after removing duplicates and excluding citations before January 2017 or identified in the previous review, of which 15 studies fulfilled the inclusion criteria and are described in Figure 1 indicated below.


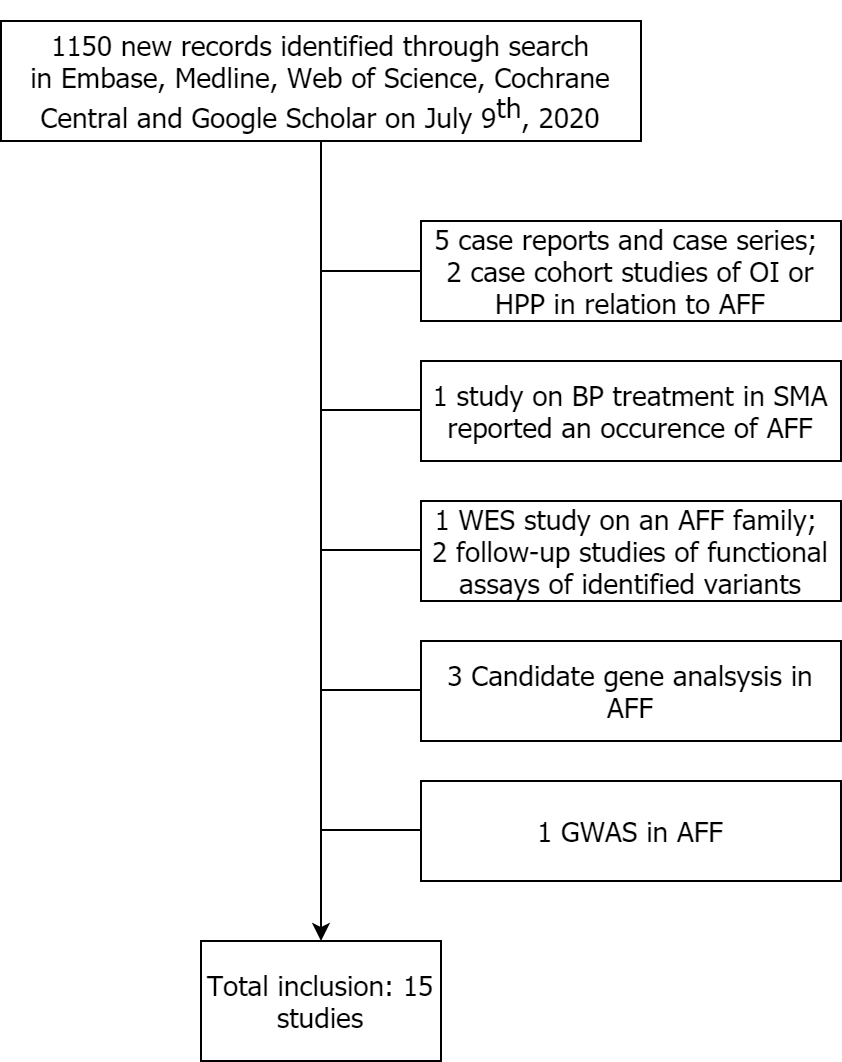


**Figure 1.** Flow diagram of search results. AFF, atypical femur fracture; OI, osteogenesis imperfecta; HPP, hypophosphatasia; BP, bisphosphonate; SMA, spinal muscular atrophy; GWAS, genome-wide association study.

**Abbreviations of genes and proteins**

*ALPL*, alkaline phosphatase

*ATR*, ATR serine/threonine kinase

*ZBTB4*, zinc finger and BTB domain containing 4

*ATRAID*, all-trans retinoic acid induced differentiation factor

*CNTN4*, contactin 4

*COL1A1*, collagen type I alpha 1 chain

*COL1A2*, collagen type I alpha 2 chain

*CRTAP*, cartilage associated protein

*CTSK*, cathepsin K

*CYP1A1*, cytochrome P450 family 1 subfamily A member 1

*FN1*, fibronectin 1

*GALNS*, galactosamine (N-Acetyl)-6-sulfatase

*GGPS1*, geranylgeranyl diphosphate synthase 1

GGPPS, geranylgeranyl pyrophosphate synthase

*IFITM5*, interferon induced transmembrane protein 5

*IL18R1*, Interleukin 18 Receptor 1

*LRP5*, LDL receptor related protein 5

*MVD*, mevalonate diphosphate decarboxylase

*NR3C1*, nuclear receptor subfamily 3 group C member 1

*NTN1*, netrin 1

*OFD1*, centriole and centriolar satellite protein

*PHEX*, phosphate regulating endopeptidase homolog X-linked

*PLS3*, plastin 3

*RUNX2*, runt related transcription factor 2

*SERPINF1*, serpin family F member 1

*SLC37A3*, solute carrier family 37 member 3 gene

*SMN1*, survival of motor neuron 1, telomeric

*TUBB8P5*, tubulin beta 8 class VIII pseudogene 5
